# MitoCore: a curated constraint-based model for simulating human central metabolism

**DOI:** 10.1186/s12918-017-0500-7

**Published:** 2017-11-25

**Authors:** Anthony C. Smith, Filmon Eyassu, Jean-Pierre Mazat, Alan J. Robinson

**Affiliations:** 1 Medical Research Council Mitochondrial Biology Unit, University of Cambridge, Cambridge Biomedical Campus, Hills Road, Cambridge, CB2 0XY UK; 2 Institute of Biochemistry and Genetics of the Cell, CNRS-UMR5095, 1 Rue Camille Saint Saëns, 33077 Bordeaux cedex, France; 3 University Bordeaux Segalen, 146 rue Léo Saignat, 33076 Bordeaux cedex, France

**Keywords:** Constraint-based model, Metabolic network, Flux balance analysis, Central metabolism, Mitochondria, Mitochondrial metabolism

## Abstract

**Background:**

The complexity of metabolic networks can make the origin and impact of changes in central metabolism occurring during diseases difficult to understand. Computer simulations can help unravel this complexity, and progress has advanced in genome-scale metabolic models. However, many models produce unrealistic results when challenged to simulate abnormal metabolism as they include incorrect specification and localisation of reactions and transport steps, incorrect reaction parameters, and confounding of prosthetic groups and free metabolites in reactions. Other common drawbacks are due to their scale, making them difficult to parameterise and simulation results hard to interpret. Therefore, it remains important to develop smaller, manually curated models.

**Results:**

We present MitoCore, a manually curated constraint-based computer model of human metabolism that incorporates the complexity of central metabolism and simulates this metabolism successfully under normal and abnormal physiological conditions, including hypoxia and mitochondrial diseases. MitoCore describes 324 metabolic reactions, 83 transport steps between mitochondrion and cytosol, and 74 metabolite inputs and outputs through the plasma membrane, to produce a model of manageable scale for easy interpretation of results. Its key innovations include a more accurate partitioning of metabolism between cytosol and mitochondrial matrix; better modelling of connecting transport steps; differentiation of prosthetic groups and free co-factors in reactions; and a new representation of the respiratory chain and the proton motive force. MitoCore’s default parameters simulate normal cardiomyocyte metabolism, and to improve usability and allow comparison with other models and types of analysis, its reactions and metabolites have extensive annotation, and cross-reference identifiers from Virtual Metabolic Human database and KEGG. These innovations—including over 100 reactions absent or modified from Recon 2—are necessary to model central metabolism more accurately.

**Conclusion:**

We anticipate MitoCore as a research tool for scientists, from experimentalists looking to interpret their data and test hypotheses, to experienced modellers predicting the consequences of disease or using computationally intensive methods that are infeasible with larger models, as well as a teaching tool for those new to modelling and needing a small, manageable model on which to learn and experiment.

**Electronic supplementary material:**

The online version of this article (10.1186/s12918-017-0500-7) contains supplementary material, which is available to authorized users.

## Background

Human central metabolism is a large and complex system under sensitive homeostatic control, and its disturbance is causative or associated with many diseases and responses to toxins. However, it is often difficult to relate more than a handful of these changes to their underlying origin or their down-stream impact, due to the highly connected nature of the reactions of central metabolism. Computer models are widely accepted in many fields as a tool to incorporate complexity and simulate changes, allowing predictions to be made and providing a unifying framework to interpret empirical data, especially from large, noisy and incomplete data sets. Yet modelling is treated with scepticism by many biomedical researchers despite their potential broad utility [1]. Simple models of enzyme kinetics (using the assumptions of Henri-Michaelis-Menten kinetics [2]) are familiar to biomedical scientists, but are impractical for simulations of central metabolism due to every reaction needing parameterisation, alongside the computational expense of solving the large set of differential equations. However, constraint-based models of metabolism used in conjunction with methods such as flux balance analysis [3] are particularly useful for simulating metabolic changes in large metabolic networks as they can incorporate flexibility, do not require kinetic parameters and are computationally inexpensive. Many genome-scale constraint-based models [4–10] have covered central metabolism and used successfully to model diseases [11, 12]. But these models do not simulate the realistic production rate of ATP (with the recent exception of Recon 2.2 [10]), a crucial element of modelling central metabolism. Furthermore, the interpretation of simulation results from thousands of reactions is difficult (especially for new-comers). In addition, attempts to simulate diseases can result in the prediction of physiologically improbable reaction fluxes due to erroneous “short-circuits” and energy-generating cycles [13]. These are caused by several common problems including: incorrect parameters for directionality constraints, the assignment of reactions to the wrong cellular compartments, or inaccurate representations of pathways, enzymes, transport steps, prosthetic groups and metabolites. These errors can introduce unrealistic bypasses and shuttles that appear to compensate for a disease state. For example, proton-coupled mitochondrial transporters running in reverse and thus pumping protons that contribute to ATP generation by the mitochondrial ATP synthase, and the confounding of free co-factors with prosthetic groups, especially the flavin adenine dinucleotide of mitochondrial succinate dehydrogenase (SDH) and electron-transferring flavoprotein (ETF), leading to incorrect electron transport between isolated complexes mediated by bound FAD/FADH. These problems are common in genome-scale models that include an initial auto-generation of the reaction network from databases that can include incomplete or incorrect annotation. These issues are particularly acute for modelling mitochondrial metabolism and metabolite transport, as all the current genome-scale models neglect the electrical gradient component (ΔΨ) of the proton motive force (PMF), and the correct proton cost of making ATP by the mitochondrial ATP synthase in animals [14]. It is also sometimes questionable whether the enormous size and complexity of these genome-scale models benefits simulations where only subsystems of cellular metabolism are of interest, such as central metabolism. Furthermore, the scale of genome-scale models can make some techniques computationally infeasible, such as elementary mode analysis [15], and the longer runtimes for their simulations and the complexity of the results can hinder exploratory analyses and hypothesis testing.

Many problems of genome-scale models can be avoided by using smaller, curated models validated against data from normal and disease metabolism. A more focussed and carefully defined model allows the user to examine and be confident in each reaction, and more clearly elucidate the behaviour of the system, including any short-comings, as well as interpret the results more easily. These ‘core’ models have been shown to be useful in a range of areas [16, 17] and we previously applied this approach for our *i*AS253 model of the mitochondrion, which we used to simulate metabolic diseases of the tricarboxylic acid cycle [18]. This model was then used as a basis to simulate other disorders including hypoxia during cardiac ischemia [19], fumarate hydratase deficiency [20] and common diseases of the mitochondrial electron transport chain [21]. These simulation results were used to generate detailed mechanistic hypotheses for data interpretation and to design further experiments. However, we recognised that this model could be improved upon by constructing a new model encompassing more of central metabolism and explicitly modelling physiochemical features such as the mitochondrial proton motive force. In particular, ease-of-use could be improved by providing extensive annotation of reactions and their parameters.

Here we present MitoCore, a new constraint-based model of central metabolism that addresses these issues and comprehensively expands upon and refines our previous mitochondrial models. This model has been designed to be easy-to-use, includes extensive annotation, has default parameters to simulate human cardiomyocyte metabolism, and is encoded in the widely used SBML format [22]. We anticipate the model will be of great use to those wishing to interpret empirical data by comparing it to simulations of central metabolism and thus investigate predictive models of disease and toxicology.

## Results

### Building the MitoCore model of human central metabolism

We designed MitoCore as a constraint-based model of central metabolism with two compartments; one representing the cytosol, outer mitochondrial membrane, inter-membrane space and cytosolic side of the inner mitochondrial membrane, and the other the mitochondrial side of the inner membrane and the mitochondrial matrix. The accuracy and utility of a metabolic model is dependent upon the reactions included and the correct partitioning of these reactions between compartments. Thus to create MitoCore, we built a list of candidate reactions to include by considering human reactions in the KEGG [23], HumanCyc [24] and BRENDA [25] databases that use any metabolites involved in central metabolism, and assigned each reaction to the appropriate cellular compartment(s) by assessing the localisation evidence collated in the MitoMiner database [26] for its catalysing protein. For reactions catalysed by enzymes with a large amount of evidence for mitochondrial localisation but lacking specific evidence for being in the mitochondrial matrix or matrix side of the inner membrane, we applied the principle of metabolite availability [18]. A summary of this localisation evidence is provided in the mitochondrial evidence section of the supplementary annotation file (Additional file 1, ‘Reaction & Fluxes’ worksheet) and consists of confidence scores from the MitoCarta 2 [27] inventory of genes that encode mitochondrial proteins. Reaction directionality was assigned by taking the consensus from annotation in metabolic databases, estimates of Gibbs free energy [28, 29], and general rules of irreversibility [30]. For each reaction extensive additional annotation was recorded including the original KEGG identifiers, EC number, description, gene mappings (both HUGO gene symbol and Ensembl identifiers), and evidence for the gene’s expression in heart and the protein’s mitochondrial localisation.

The partitioning of metabolism between the mitochondrion and the cytosol logically led us to consider how to model the proton motive force (PMF) and the role of protons crossing the inner mitochondrial membrane as part of oxidative phosphorylation, which produces the majority of cellular ATP. The PMF is achieved by complexes I, III and IV pumping matrix protons across the mitochondrial inner membrane and into the intermembrane space. This proton-pumping creates a proton motive force (PMF) across the membrane that has two components: a proton gradient (ΔpH) coupled with an electrical membrane potential (ΔΨ). The energy for proton-pumping comes from the transfer of electrons down the respiratory chain from NADH and ubiquinone to oxygen, to form water. Additional electrons are passed into the respiratory chain from the TCA cycle by complex II, and from the degradation of fatty acids and amino acids by the electron-transfer flavoprotein (ETF). Mitochondrial ATP synthase uses the PMF to power ATP synthesis from ADP and phosphate by channelling protons back across the inner mitochondrial membrane. It is thus clear that it is necessary to distinguish as Peter Mitchell did [31], the protons involved in chemical reactions taking place in an isolated compartment (“scalar protons”), from the protons crossing between compartments (“vectorial protons”). Therefore, to represent the PMF and distinguish between scalar and vectorial protons, we modeled the PMF in MitoCore as a metabolite that is co-transported in steps that transport charged metabolites or protons across the inner mitochondrial membrane. We accounted for the relative contributions of both ΔΨ and ΔpH to the overall PMF by co-transporting 0.82 PMF metabolites for transport steps that affect ΔΨ, and 0.18 PMF metabolites for transport steps that affect ΔpH. These values are the average of published figures of the relative contributions of ΔpH and ΔΨ by several authors (see Additional file 2 for details and references). Therefore, the reactions that represent complexes I, III and IV of the respiratory chain move PMF metabolites that correspond to the number of protons they pump from the matrix to the cytosol, as a proton in this case affects both ΔΨ and ΔpH. For example mitochondrial ATP synthase needs to transport 2.7 PMF metabolites back to the matrix to synthesise one molecule of ATP (as it uses 2.7 protons per molecule of ATP [14]). We modelled electrogenic and proton-coupled transport steps between the two compartments in the same way; for example the mitochondrial ATP/ADP carrier 1 (SLC25A4) requires 0.82 PMF to be co-transported with each imported ADP^3−^ and exported ATP^4−^ nucleotides to reflect the charge difference that affects only ΔΨ (Eq. 1), whereas the proton-coupled phosphate carrier (SLC25A3) imports 0.18 PMF as overall transport is electro-neutral and so only affects ΔpH (Eq. 2):

1 $$ {\mathrm{ATP}}_{\mathrm{mito}}+{\mathrm{ADP}}_{\mathrm{cytosolic}}+0.82\ {\mathrm{PMF}}_{\mathrm{cytosolic}}\rightarrow {\mathrm{ATP}}_{\mathrm{cytosolic}}+{\mathrm{ADP}}_{\mathrm{mito}}+0.82\ {\mathrm{PMF}}_{\mathrm{mito}} $$

2 $$ {{\mathrm{H}}^{+}}_{\mathrm{cytosolic}}+0.18\ {\mathrm{PMF}}_{\mathrm{cytosolic}}+{\mathrm{Pi}}_{\mathrm{cytosolic}}\kern0.5em \rightarrow {{\mathrm{H}}^{+}}_{\mathrm{mito}}+0.18\ {\mathrm{PMF}}_{\mathrm{mito}}+{\mathrm{Pi}}_{\mathrm{mito}} $$

We next modelled the remaining transport steps that connect the compartments, and the influx and efflux of metabolites to the cytoplasm. We defined four different transport categories. First were transport steps based on the characterised mitochondrial transport proteins. Many carriers can transport a range of related substrates (although with different affinities) and we modelled each metabolite combination as a separate step including counter exchange and proton-coupling. In some cases proton-coupled transporters were represented by two reactions to model separately the forward and reverse directions, and a proton (plus co-transported PMF metabolite) only used for movement down the proton gradient. This prevented transporters being used in reverse to pump protons artificially (and so transferring PMF) and thereby unrealistically contributing to ATP production by the mitochondrial ATP synthase. The second category was for metabolites whose transporters are unidentified. We modelled these steps as uniporters that are not proton coupled. If the metabolite was charged and moving into the mitochondrial matrix we assumed this would impact ΔΨ and co-transported 0.82 PMF metabolite accordingly. The third category was for metabolites that can diffuse across the inner mitochondrial membrane—including oxygen, carbon dioxide and water—and these were modelled as reversible uniport transport steps. Finally we modelled the insertion of lipids into the mitochondrial inner membrane via the flippases, which was coupled to ATP hydrolysis.

To account for the generation of reactive oxygen species (ROS) from the respiratory chain in MitoCore, we defined their production as 0.001% of the flux through complex I—the primary site of ROS generation from the respiratory chain—and hence also reduce the efficiency of proton-pumping [32, 33]. Subsequent reactions model ROS being converted to water at the expense of NADPH.

Various reactions defined in KEGG do not distinguish between free and prosthetic redox cofactors, e.g. FAD/FADH, which can lead to unrealistic electron transport being mediated between prosthetic redox co-factors. To prevent this in MitoCore, we removed prosthetic co-factors from reactions (such as for complex II and the ETF), and instead directly coupled their reactions, e.g. to the reduction of ubiquinone to ubiquinol.

For convenience in setting up simulations, we created four ‘pseudo’ reactions that summarise aspects of MitoCore’s biological activity and can be used by flux balance analysis as objective functions: ATP hydrolysis (representing cellular ATP demand), and the biosynthesis of heme, lipids and amino acids.

To enable comparison of results from MitoCore to those of genome-scale models such as Recon 2 [5], we re-used identifiers for metabolites and reactions present in the Virtual Metabolic Human database (https://vmh.uni.lu) for MitoCore where possible. However, it was necessary to create 105 new reactions for MitoCore (Additional file 3) that were either absent from the Virtual Metabolic Human database and Recon 2.2 [10] (such as transport steps or compartment specific versions), inaccurately described (such as specifying prosthetic FAD as a free co-factor), or needed to represent new features (such as the proton motive force). New reaction identifiers were appended with the suffix ‘MitoCore’. Pathways represented in MitoCore include glycolysis, pentose phosphate pathway, TCA cycle, electron transport chain, synthesis and oxidation of fatty acids, ketone body and amino acid degradation and cover all parts of central metabolism involved directly or indirectly with ATP production. Finally, MitoCore was extensively tested to ensure: it contained no erroneous energy-generating cycles; its simulation results were consistent with disorders we investigated previously, such as ischemia and mitochondrial diseases [18–21]; each reaction was capable of having a flux (depending on the constraints placed on the cytosolic boundary transport steps) and so ensure it contained no reaction dead-ends.

To enable others to follow our reasoning for assigning reactions to specific compartments and their directionality, we recorded the provenance for reactions (Additional file 1). For example, the directionality evidence section describes why a constraint has been set; a manually evaluated consensus of information from the KEGG [23], HumanCyc [24] and BRENDA [25] databases, general rules of irreversibility [30], large ΔG values from eQuilibrator [29] or estimated using a group contribution method [28], and information from the literature. In cases for which reaction directionality was unclear, it was kept reversible. To address why a reaction has been included in our cardiomyocyte model, we included a heart expression section consisting of RNAseq and immunochemistry expression levels of genes taken from the Human Protein Atlas (version 14) [9]. The spreadsheet also includes gene mappings, identifiers from Recon 2 and KEGG, mitochondrial localisation evidence (as described above) and baseline reaction fluxes when the objective function was maximum ATP production under normal conditions. For distribution, we encoded MitoCore in SBML [22] (Additional file 4) and produced a companion annotation Excel spreadsheet (Additional file 1).

### Simulating cardiomyocyte metabolism using MitoCore and flux balance analysis

**Fig. 1:**
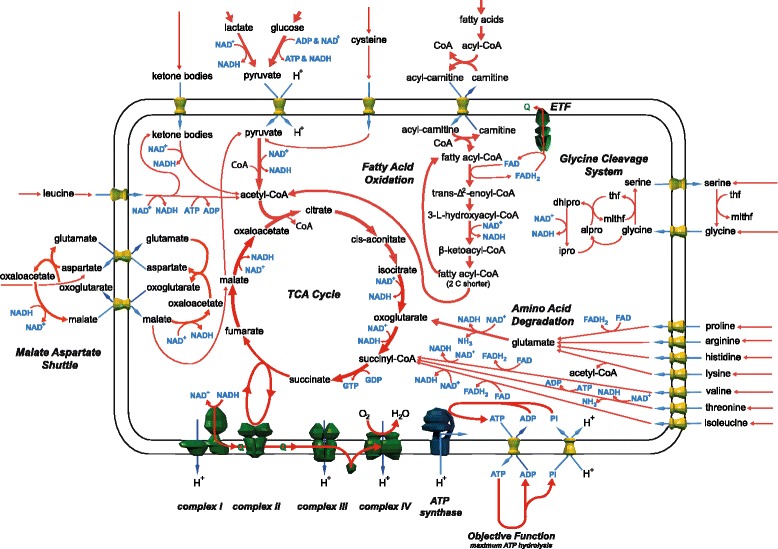
Summary of the major active pathways of central metabolism in the flux balance analysis simulation of the MitoCore model with default parameters and the objective function of maximum ATP production. Values of all fluxes are reported in Additional file 1.

MitoCore’s default reactions and parameters are optimised for cardiomyocytes and use the metabolites available to healthy hearts of glucose, fatty acids, ketone bodies and amino acids (references listed in Additional file 1). To demonstrate MitoCore’s capability of producing physiological relevant results using these parameters, we simulated cardiomyocyte metabolism by using flux balance analysis (FBA) [3]. To reflect the primary role of central metabolism in cardiomyocytes we set the simulation’s objective as maximum ATP production and calculated the optimum reaction fluxes through central metabolism. The resultant reaction fluxes simulated core metabolism correctly with activity of all respiratory complexes, the TCA cycle and malate-aspartate shuttle (Fig. 1, Additional file 1). As metabolic fuels were provided in slight excess, the availability of oxygen limited the overall fluxes. Simulated ATP production was 100.9 μmol/min/g of dry weight. Sources of acetyl-CoA for the TCA cycle were fatty acid degradation (55.0%), glucose oxidation (26.4%), lactate oxidation (8.4%), ketone body degradation (6.1%), amino acid degradation (3.8%) and glycerol oxidation (0.3%). The amino acids degraded and used to produce ATP were histidine, isoleucine, leucine, lysine, threonine, valine, arginine, aspartate, cysteine, glycine, proline, serine, asparagine, and alanine. Ammonia, produced as a by-product of amino acid degradation, was exported from the system. To determine the robustness of the FBA simulation results with these parameters, we performed Flux Variable Analysis (FVA) at 100% and 98% of the optimal solution (See Methods and Additional file 1).

### Metabolite degradation and ATP yields with MitoCore and Recon 2.2

To demonstrate how different metabolites are degraded appropriately in the MitoCore model and the effect on ATP production of implementing the proton motive force, we performed a series of simulations using particular ‘fuel’ metabolites in isolation. We simulated the oxidation and degradation of glucose, lactate, hexadecanoic acid, hydroxybutanoate, acetoacetate and 20 different amino acids in separate simulations (Additional file 5). The simulations showed that in each case the degradation routes used were biologically plausible. Unsurprisingly fatty acids were the most energy rich fuels (ATP production of 112 μmol/min/g of dry weight), followed by the amino acids tryptophan (43 μmol/min/g of dry weight), isoleucine (38 μmol/min/g of dry weight), leucine (37 μmol/min/g of dry weight) and phenylalanine (36 μmol/min/g of dry weight) and then glucose (33 μmol/min/g of dry weight).

To compare the simulations by MitoCore with those from a genome-scale model, we performed the same metabolite degradation and ATP yield simulations with the human genome-scale model Recon 2.2 [10] (Table 1). The Recon 2.2 model used biological plausible routes in the degradation of many metabolites, such as histidine, isoleucine, leucine, lysine, phenylalanine, threonine, tryptophan, valine, arginine, cysteine, glutamate, glutamine, glycine, and tyrosine. However, non-canonical pathways were used with several other metabolites. For example, in glucose metabolism, Recon 2.2 produced lactate from pyruvate, transported this lactate into the mitochondrion and then converted it back to pyruvate, bypassing both the pyruvate carrier and the malate-aspartate shuttle. Lactate degradation followed a similar route. For fatty acid degradation, the Recon 2.2 simulation used summary reactions for β-oxidation (instead of the individual degradation steps that are also present in the model) that have FADH as a cofactor. The electrons from this FADH then incorrectly entered the electron transport chain via the prosthetic FADH of one of the reactions representing mitochondrial succinate dehydrogenase (SDH), also resulting in the two reactions representing the activity of the SDH complex not having the same flux. In addition, the carnitine shuttle was inactive in Recon 2.2 simulations. In some cases, the degradation of particular amino acids used reactions that are unlikely to be present in humans, such as 3-hydroxypropionate dehydrogenase (EC 1.1.1.59, Recon 2.2 reaction ID: r0365) used in methionine degradation, or used a reaction in a direction that is unlikely in vivo, such as for pyrroline-5-carboxylate reductase 1 (EC 1.5.1.2, Recon 2.2 reaction ID: PRO1xm) in proline degradation.

**Table 1:** Comparison of maximum ATP yields from different carbon source ‘fuel’ metabolites

Metabolite	MitoCore ATP Yield	Recon 2.2 ATP Yield
Glucose	33.0	32.0
Fatty acid hexadecanoate (C16)	111.8	106.8
Lactate	15.5	15.0
Hydroxybutanoate	23.1	20.3
Acetoacetate	20.2	18.0
Histidine	26.3	25.5
Isoleucine	37.8	35.5
Leucine	37.1	33.5
Lysine	36.2	32.0
Methionine	18.3	27.0
Phenylalanine	36.2	33.0
Threonine	20.1	19.0
Tryptophan	43.1	39.5
Valine	30.1	29.0
Arginine	29.2	27.5
Aspartate	16.0	15.0
Cysteine	16.1	17.5
Glutamate	23.8	22.5
Glutamine	24.0	22.5
Glycine	8.0	8.0
Proline	28.1	27.5
Serine	13.1	13.5
Tyrosine	38.9	35.5
Asparagine	16.0	15.0
Alanine	16.0	15.0

### Simulating the mitochondrial disease fumarase deficiency with MitoCore and Recon 2.2

To compare the ability MitoCore and Recon 2.2 to model diseases of central metabolism, we performed preliminary simulations of the mitochondrial disease fumarase deficiency (OMIM:606812). Fumarate deficiency is a mitochondrial disease resulting from a defect in the fumarate hydratase gene (ENSG00000091483) that encodes both cytosolic and mitochondrial isoforms, and that converts fumarate to malate—a key step in the TCA cycle. This can leave patients with almost no residual enzyme activity and causes developmental delay, severe mental retardation, seizures and dysmorphic facial features [34–37]. A key diagnostic marker for the disease is the presence of fumarate in the cerebrospinal fluid.

**Fig. 2:**
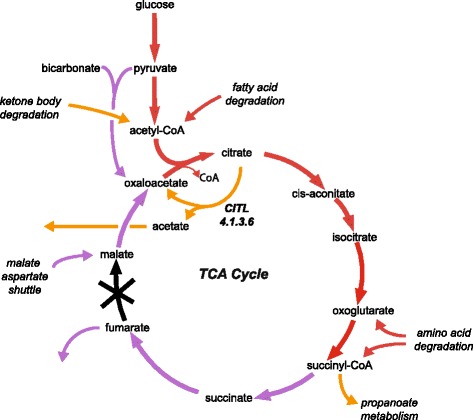
Summary of the compensatory pathways used by the MitoCore and Recon 2.2 models when simulating fumarase deficiency with the objective function of maximum ATP. (Red arrows represent reactions active in both models; purple arrows are only active in the MitoCore model; and orange arrows are only active in the Recon 2.2 model)

To simulate the disease in each model we used the default cardiomyocyte uptake parameters of MitoCore (replicating them in Recon 2.2) and inactivated both the cytosolic and mitochondrial reactions that represent the enzyme. The MitoCore model showed a 72% reduction in ATP production upon deletion of fumarate hydratase compared to the default model (Fig. 2, Additional file 6). Flux through the TCA cycle continued at a lower level until reaching fumarate, which was effluxed. To bridge the break in the TCA cycle due to the inactive fumarate hydratase and allow the TCA cycle to complete, oxaloacetate was produced from pyruvate (by using mitochondrial pyruvate carboxylase) and the malate-aspartate shuttle and fed into the TCA cycle. Amino acid degradation further bolstered flux through the TCA cycle. All these compensatory reactions seemed biologically plausible. In contrast, Recon 2.2 showed a much lower drop in ATP production of 17% upon inactivation of fumarate hydratase activity. This was largely due to compensation by a reaction that represents citrate (pro-3S)-lyase (Recon 2.2 reaction ID:CITL, EC: 4.1.3.6) that allowed the degradation of fatty acids and ketone bodies to continue at a higher rate than was possible in the MitoCore model by converting citrate to oxaloacetate and acetate, which was effluxed. However, citrate (pro-3S)-lyase is absent from humans and the gene assigned to this reaction in the Recon 2.2 model has been characterised to convert glyoxylate and acetyl-CoA to malate [38].

### The effect of proton leak on the electron transport chain in MitoCore

**Fig. 3:**
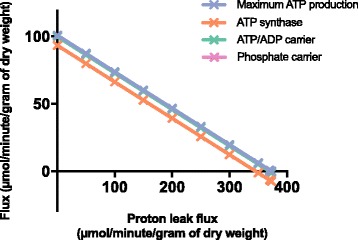
The effect of increasing proton leak through UCP2 during simulations of the MitoCore model on: maximum ATP production, flux through mitochondrial ATP synthase, flux through the mitochondrial ATP/ADP transporter, and flux through the mitochondrial phosphate transporter. The flux through ATP synthase is distinct from all the other fluxes that are superimposed, because there is also synthesis of mitochondrial ATP from the TCA cycle

To show the MitoCore model can simulate experimentally induced conditions that are directly affected by the proton motive force, we performed a series of simulations representing an increasing proton leak across the mitochondrial inner membrane. We introduced a flux through the transport step that represents the leak of matrix protons to the cytosol through the uncoupling protein 2 (UCP2) in cardiomyocytes [39]. The optimum reaction fluxes for maximum ATP production (Additional file 7) were identical to those under default constraints with the exception of the steps involved in mitochondrial ATP synthesis. Maximum ATP production was reduced as the proton leak increased due to the progressively reduced flux through mitochondrial ATP synthase until it reversed under very high leak (Fig. 3), and used ATP synthesized in the TCA cycle. At the highest level of proton leak the flux also reversed through the mitochondrial ATP/ADP carrier and the phosphate carrier to import additional ATP from glycolysis.

## Discussion

Here we present MitoCore, a curated, constraint-based model of human central metabolism, designed as a predictive model of metabolism in disease and toxicology, and for use by a wide range of researchers. It covers all major pathways involved in central metabolism using 407 reactions and mitochondrial transport steps, and 74 transport steps over the plasma membrane. To increase the metabolic flexibility of MitoCore, we included a large number of reactions that were not assigned to classical metabolic pathways, but could have potentially important roles in supporting central metabolism. MitoCore was parameterised and annotated for cardiomyocyte metabolism, which is useful for many types of analyses as the cardiomyocyte can metabolise a wide range of substrates and has reactions common to many other cell types, as well as representing the metabolism of an organ of utmost importance in human health, disease and toxicology. This allows the simulation results to be generalisable, without having features that are particularly cell specific, such as those found only in hepatocytes. However, we have included reactions that are inactivated in the default cardiac model, but can be activated to represent the metabolic capabilities of other cell types, e.g. gluconeogenesis, ketogenesis, β-alanine synthesis and folate degradation. Thus, the model allows biologically relevant flux distributions to be generated ‘out of the box’ without altering the model, while allowing for easy modification to represent metabolism in other cell types.

MitoCore has several unique features. The first is the more accurate partitioning of metabolism between the mitochondrion and the cytosol by using extensive localisation data and annotation. This partitioning has an important impact on model behaviour as the limited transport steps into the mitochondrial matrix result in dramatic differences in metabolite availability in the matrix compared to the cytosol. Therefore, it is important that reactions are assigned to the correct compartment. We achieved this by manually evaluating the subcellular localisation of each reaction’s catalysing protein by using the mitochondrial localisation evidence in the MitoMiner database [26].

To model oxidative phosphorylation in MitoCore, we devised a new representation of the PMF and mitochondrial respiratory chain—the second unique feature of MitoCore. MitoCore’s representation of the respiratory chain differs in many key aspects to other metabolic models, due to how it models vectorial protons and accounts for both components of the PMF. MitoCore represents the PMF as a metabolite that is co-transported in steps that transport charged metabolites or protons across the inner mitochondrial membrane, such as the reactions of the respiratory complexes, and in proton-coupled and electrogenic transport steps. This separate modelling of vectorial protons using an additional new PMF metabolite enabled the impact of electrogenic transporters on the PMF to be accounted for in flux balance analysis simulations for the first time, and prevented simulation artefacts where (scalar) protons generated or removed from other parts of metabolism allow unrealistically high ATP production.

The third unique feature of MitoCore is the physiochemical modelling of the transport steps that connect the cytosolic and mitochondrial compartments including their impact on the PMF, and the influx and efflux of metabolites over the plasma membrane. 83 transport steps connect the two compartments, of which 30 are modelled on the known transport mechanisms of characterised transport proteins of the inner mitochondrial membrane, whereas the other 53 represent known transport capabilities of the membrane, such as diffusion of small metabolites. 74 transport steps at the cytosolic boundary represent the import and export of metabolites across the plasma membrane, such as oxygen, carbon dioxide, glucose, fatty acids and amino acids.

MitoCore improves over other models by distinguishing between free and prosthetic redox co-factors. This keeps separate the electrons entering the respiratory chain from different sources, which can otherwise become connected via e.g. a shared flavin adenine nucleotide metabolite, and is particularly relevant under perturbed conditions where the erroneous connection of free and prosthetic flavin adenine nucleotides can cause unrealistic electron transport bypasses to occur. This problem is endemic in large-scale models that auto-generate reaction networks directly from metabolic databases without manual curation of reactions.

Other improvements to MitoCore included modelling protein complexes as one reaction rather than a series of linked reactions, as can often be found in metabolic pathway databases and other models. This is particularly important for simulations of gene knockouts, as a gene deficiency will disrupt the activity of the whole protein complex rather than just one of its subunit’s reactions. Further, to facilitate gene-based analyses (such as knock out studies) we provide reaction-to-gene mappings with both gene symbols and Ensembl identifiers. Finally, to summarise different aspects of central metabolism during simulations with MitoCore, we defined four ‘pseudo’ reactions for biosynthesis of amino acids, lipids and heme that are required by cells for maintenance and growth, and ATP hydrolysis to reflect the energy demand of the cell. These pseudo reactions are designed to be used as objective functions during flux balance analysis and can determine if particular functions of central metabolism have become impaired during simulations of disease.

A weakness when it comes to interpreting many metabolic models is the lack of provenance about their components. For example recording why reactions have been included, why directionality constraints have been set, or the origin of reaction parameters. Thus for MitoCore we created a supplementary annotation spreadsheet (Additional file 1) to record this provenance. The spreadsheet also serves as a useful template to map flux distributions onto, as reaction fluxes can be grouped in an intuitive way directly against useful supplementary information. When combined with the small size of the model, simulations can be generated quickly and then easily interpreted.

To demonstrate that MitoCore produces physiologically realistic results, we simulated cardiomyocyte metabolism using the default parameters with flux balance analysis [3] and the objective of maximum ATP production. The reaction fluxes showed central metabolism was modelled correctly, with the largest fluxes through the TCA cycle and respiratory chain. Numerous fuel sources (fatty acids, glucose, lactate, ketone bodies and amino acids) were imported, degraded and entered the TCA cycle at several different points (Fig. 1). The combination of different fuel metabolites used in the simulations showed similar trends to experimental ranges in well-perfused heart (although the reported ranges are large, reflecting the wide range of metabolites that the heart can potentially use). For example, experimental measurements of the sources of acetyl-CoA for the TCA cycle [40] report the majority of acetyl-CoA (60–90%) derives from fatty acids, which is close to the 55% in the MitoCore simulation, whereas glycolysis (including lactate oxidation) accounts for 10–40% of acetyl-CoA compared to 35% in the MitoCore simulation, with the remainder of acetyl-CoA derived from amino acids. Finally, to show the robustness of the simulation results, we performed Flux Variability Analysis (FVA). The ranges for reaction fluxes calculated (Additional file 1) showed a realistic range of values and a lack of flux loops (apart from interrelated mitochondrial transport steps), with most of the fluxes uniquely determined or having a limited range with large non-fixed fluxes corresponding to isoenzymes or transport steps whose fluxes exchange while maintaining a fixed total flux.

To show the importance of explicitly modelling the PMF, we simulated the maximum ATP production achievable using 1 μmol/min/g of dry weight of common metabolic fuels in isolation (effectively calculating their ATP yields) and compared the results to those generated by the genome-scale model Recon 2.2 [10] and from theoretical calculations [41] (Table 1 and Additional file 5). Recon 2.2 is an update of the widely used Recon 2 model [5], which also separates protons used by the electron transport chain and the aspartate-glutamate carrier from the rest of metabolism to allow it to predict ATP yields, unlike its predecessor, Recon 2, that produces infinite ATP under these conditions [10]. However, Recon 2.2 does not directly capture the energetic cost on ATP production of other proton coupled mitochondrial transport steps or the ΔΨ part of the PMF. In MitoCore each glucose produced 33 ATP in comparison to 32 calculated theoretically [41] and 32 in Recon 2.2 [10]. For the fatty acid hexadecanonic acid 112 ATP were produced compared to 108 theoretically [41] and 107 in Recon 2.2. A significant difference between the models is the number of protons required by mitochondrial ATP synthase to produce one molecule of ATP: MitoCore uses 2.7 (based on the structure of the bovine mitochondrial ATP synthase [14]) whereas the theoretical calculations use 2.5 and Recon 2.2 uses 4.0 (presumably to account indirectly for the electroneutral proton-coupled phosphate carrier and electrogenic charge-coupled ATP/ADP exchange). Due to also considering additional factors that affect ATP production—including the impact and bioenergetic cost of all the transport steps on the PMF as well as ROS production and removal—we believe our figure is likely to be more accurate than both Recon 2.2 and these theoretical calculations. When comparing the flux distributions between MitoCore and Recon 2.2 it appears that despite having similar ATP yields for many metabolites, the pathways used by MitoCore are more biologically reasonable for some metabolites (i.e. using canonical degradation pathways) and lacked some of the unlikely elements found in Recon 2.2, such as using a lactate shuttle instead of the pyruvate carrier during glucose oxidation. In some Recon 2.2 simulations the malate-aspartate shuttle was inactive, presumably due to its use having a direct penalty on ATP production, the only transport step defined in Recon 2.2 to do so. MitoCore avoids this problem of unrealistic bypasses by using PMF penalties on all the relevant mitochondrial transport steps.

Further differences could be seen when comparing the results of preliminary simulations of fumarase deficiency (Fig. 2) that showed MitoCore replicating the efflux of fumarate seen in patients while also using plausible compensatory mechanisms, which would provide a promising starting point for further simulations and analysis (such as determining the effect of varying parameters). Recon 2.2 did not replicate the efflux of fumarate, and ATP production was maintained by using a reaction (CITL, a non-ATP dependent citrate lyase) that instead effluxed acetate. This reaction is unlikely to be present in human, which suggests the Recon 2.2 model would require further investigation and changes to simulate fumarase deficiency realistically.

An important aspect of modelling is exploring different hypotheses by running multiple simulations with different parameters. This is especially true when simulating mechanisms of disease, which may reveal compensatory mechanisms and the effect of supplying different metabolites to compensate for metabolite deficiencies. Therefore, it is beneficial if simulation runtimes are comparatively short. Even when using computationally expensive types of flux balance analysis, such as geometric FBA, MitoCore takes ~10 s per run, compared to ~3 h per run for Recon 2.2. The short runtime for MitoCore simulations makes the exploration of varying parameters much easier and routine, computationally expensive methods feasible, and allows rapid testing of new ideas and hypotheses.

When comparing maximum ATP production using default parameters to the previous *i*AS253 model using the same parameters [18], ATP production was notably lower (101 vs 140 μmol/min/g of dry weight) due to the introduction of PMF metabolites in the electron transport chain and transport steps, demonstrating it has an important effect. The results from the proton leak simulations (Additional file 7) show the use of metabolites to represent the PMF enables the model to replicate experimental observations under perturbed conditions that would otherwise be impossible, such as the reversal of mitochondrial ATP synthase, the ATP/ADP carrier and the phosphate carrier. Such behaviour is well-known in the absence of respiratory chain activity, for instance in ρ^0^ cells to maintain a proton motive force in mitochondria [42, 43].

Taken together these simulation results show the MitoCore model is capable of producing realistic results using a wide range of metabolites while avoiding some of the common problems of genome-scale models, including: unlikely shuttling between compartments, reactions that are not compartmentalized correctly, incorrect directionality constraints and the inclusion of reactions that are unlikely to be present in human. These problems, while understandable due to the scale of these models, nevertheless make producing biological relevant flux distributions difficult, especially under perturbed conditions, and show the continued need for carefully curated ‘core’ models for some types of simulation. Further, these comparisons illustrate an additional role for ‘core’ models, which is the ability to identify and diagnose problem areas for improvement in genome-scale models.

## Conclusions

MitoCore is a constraint-based model of human central metabolism, provided with default parameters that provide physiologically realistic reaction fluxes for cardiomyocytes in FBA simulations. The model has several innovations including a new representation of the respiratory chain and proton motive force, and partitioning of reactions to subcellular compartments based on the latest localisation evidence. To achieve an accurate depiction of central metabolism, each of MitoCore’s 407 reactions and transport steps was manually evaluated for directionality, expression of its gene in heart, and subcellular localisation of its protein. To allow MitoCore to be easily used and to make the results comparable with other models and compatible with other types of analyses, we used identifiers from the Virtual Metabolic Human database for both reactions and metabolites where possible, and recorded KEGG identifiers in the annotation. To help ease of use, we also provide an annotation spreadsheet that provides gene mappings, localisation and heart expression evidence, and notes on parameter choice. MitoCore is provided in SBML format to be compatible with a wide range of software. We hope MitoCore will be of use as a research tool to a wide range of biomedical scientists and students—from experienced modellers interested in central metabolism or using computationally intensive methods that are infeasible on a genome-scale model, to those new to modelling who would like to begin by using a small manageable model, with application as a predictive model of disease.

## Methods

### Identifying reactions of central metabolism to include in MitoCore

The starting point for the MitoCore model was an updated version of the *i*AS253 mitochondrial model [21]. The model was expanded by searching for human reactions in KEGG [23], HumanCyc [24] and BRENDA [25] that were missing from the *i*AS253 model and could impact central metabolism. Each reaction was reassessed for subcellular localisation and directionality (see below), and to ensure the reactions were occurring in most tissues including cardiac, tissue expression of their genes and proteins was verified by using the Human Protein Atlas [9].

### Partitioning reactions between the cytosol and mitochondrion in MitoCore

To partition the reactions into either the cytosol or mitochondrion, each enzyme was manually evaluated by using the mitochondrial localisation evidence in the MitoMiner database [26]. MitoMiner collates GFP tagging, large-scale mass-spectrometry mitochondrial proteomics studies and mitochondrial targeting sequence predictions, with detailed annotation from the Gene Ontology and metabolic pathway data from KEGG. MitoMiner also contains homology information allowing localisation evidence to be shared amongst species. All available experimental localisation evidence, mitochondrial targeting sequence predictions and annotation were considered, including from homologs from mouse, rat and yeasts. For reactions with strong evidence for mitochondrial localisation, but where matrix localisation is unclear, the principal of metabolite availability was applied —a reaction can only be present in a compartment if all its substrates are available and its products can be used by reactions within the same compartment [18]. Reactions residing in the mitochondrial matrix or matrix side of the mitochondrial inner membrane were assigned to the mitochondrial compartment. Transport steps were created to connect the cytosol and matrix based on the transport properties of the membrane (active transport, diffusion, etc.). Each reaction was cross-referenced with the Virtual Metabolic Human database (https://vmh.uni.lu) and used its identifiers for reactions and metabolites where possible.

### Assigning reaction directionality in MitoCore

Reaction directionality was manually evaluated for each reaction in MitoCore. The KEGG [23], HumanCyc [24] and BRENDA [25] databases were consulted, and general rules of irreversibility were taken into account (such as most reactions consume ATP rather than produce it and carbon dioxide is normally produced not consumed) [30]. The ΔG values for reactions were also considered, both calculated by eQuilibrator [29] and estimated by using the group contribution method [28], and large changes noted. If reaction directionality was conflicted or unclear, then the literature was consulted. If support for irreversibility was poor or a consensus could not be found, the reaction was assigned reversible. The information used to make each assessment was recorded in the directionality evidence column of the annotation spreadsheet (Additional file 1). Further refinement of reaction directionality was used to eliminate loops that could produce metabolites such as ATP and NADH for ‘free’, and to prevent the interconversion of NADH and NADPH, unless it had been experimentally verified.

### Defining and updating reactions in MitoCore

Modelling of the proton motive force (PMF) was improved across the mitochondrial inner membrane, a pseudo-metabolite was introduced to model the effect on the PMF of proton and electrogenic transport steps across the inner mitochondrial membrane. In total 43 reactions representing respiratory complexes as well as proton-coupled transport steps were rewritten to use this new species. Next, to prevent unrealistic bypasses between free and complex-bound prosthetic flavin adenine dinucleotides, FADH and FAD^+^ were removed from all reactions and replaced with ubiquinone and ubiquinol. Finally, to represent better the current understanding of central metabolism pathways, some reactions were rewritten, such as the generation of ROS from complex I, and the stoichiometry of the proton-pumping of the respiratory complexes. In some cases a reaction’s subcellular localisation was changed by defining mitochondrial-specific metabolite species, or new reactions were written using existing metabolite species.

To highlight where there are differences between a MitoCore reaction and the corresponding reaction in the Virtual Metabolic Human database, the MitoCore identifier used the Virtual Metabolic Human database identifier with the suffix ‘MitoCore’. (N.B. Recon 2 also uses The Virtual Metabolic Human database identifiers.)

### Testing the MitoCore model

The model was extensively tested: each reaction in the model was set as the objective function in a series of simulations to ensure all reactions were capable of having flux, with directionality constraints reevaluated for both the reaction and other members of the same pathway if this was not the case; erroneous energy-generating cycles were manually identified and removed by running a succession of simulations where ATP production was maximised and a wide range of different ‘fuel’ metabolites provided; that the model used physiologically characterised pathways under normal conditions and used plausible mechanisms under perturbed conditions.

The MitoCore model was encoded in SBML v2.1 and its validity checked with the SBML online validator [44].

### Simulating metabolism

Metabolism was simulated by using flux balance analysis (FBA), which can be summarised as calculating the reaction turnover, or fluxes (flows) of metabolites through a network of biochemical reactions assuming a pseudo steady state [3]. The fluxes through the network are constrained by the stoichiometry and directionality of the reactions, as well as flux capacity and cytosol boundary uptake ranges. Cytosol boundary transport steps model the import and export of metabolites to the cell, but the overall rate of production and consumption of metabolites is assumed to be zero, hence a pseudo steady state. A metabolic objective function is chosen for each simulation, and FBA used to calculate an optimal set of reaction fluxes that maximise this function. For simulations here, maximum ATP production (by maximising flux through the pseudo reaction of ATP hydrolysis) was used as the objective function because energy generation is a major purpose of central metabolism, particularly in cardiomyocytes. To determine the robustness of the model solutions and identify the most important reactions contributing to the optimal solution, flux variability analysis (FVA) was used. FVA calculates the flux ranges of each reaction that still give the same (maximal) value of the objective function as the optimal solution, or a fraction of it. FVA was applied at 100% and 98% of the optimal ATP production.

For simulations of ATP yield, all cytosol boundary uptake fluxes for metabolites that can be degraded to produce ATP were set to zero, oxygen was increased to 50 μmol/min/g of dry weight (so that limited oxygen availability did not affect results), while other cytosol boundary conditions were unaltered. The uptake flux of each metabolite of interest was then increased to 1 μmol/min/g of dry weight. Geometric FBA simulations were then performed with maximum ATP production as the objective function. (When Recon 2.2 simulations did not obtain convergence, the ‘epilson’ and ‘flexRel’ parameters were relaxed from the defaults.)

For simulations of fumarase deficiency, the reactions corresponding to mitochondrial and cytosolic fumarate hydratase were inactivated in each model, whilst the uptakes values were left at default values. Simulations were performed with geometric FBA and an objective function of maximum ATP production. To enable geometric FBA of the Recon 2.2 model to converge to a solution, the efflux was prevented of acetone (Recon 2.2 reaction ID: EX_acetone), tetradecanoate (Recon 2.2 reaction ID: EX_ttdca), 3-(4-hydroxyphenyl)pyruvate (Recon 2.2 reaction ID: EX_34hpp_), 4-methyl-2-oxopentanoate (Recon 2.2 reaction ID: EX_34hpp_) and bicarbonate (Recon 2.2 reaction ID: EX_hco3).

For simulations of the effect of proton leak on maximum ATP production, the lower bound of the reaction representing the gene UCP2 (MitoCore reaction ID: HtmB_MitoCore) was increased over a series of simulations, thus forcing a minimum flux–representing proton leak—through the reaction.

FBA simulations were performed by using MATLAB (Math Works, Inc., Natick, MA) and the COBRA Toolbox [45], with the linear programming solver GLPK (http://www.gnu.org/software/glpk).

## Additional files

Additional file 1: Companion annotation spreadsheet recording evidence and provenance for reactions and parameters used in MitoCore, and reaction fluxes when simulating maximum ATP production using default parameters for cardiomyocytes. (XLSX 289 kb)

Additional file 2: Experimental measurements of the relative contributions of ΔpH and ΔΨ to the proton motive force. (XLSX 11 kb)

Additional file 3: 105 new or altered reactions in the MitoCore model that differ from those in the Virtual Metabolic Human database (and used by the Recon 2.2 model). (XLSX 82 kb)

Additional file 4: MitoCore model encoded in the SBML format. (XML 776 kb)

Additional file 5: Flux distributions from the MitoCore and Recon 2.2 models for maximum ATP production with different metabolic fuels. (XLSX 445 kb)

Additional file 6: Flux distributions from MitoCore and Recon 2.2 models when simulating fumarase deficiency and maximum ATP production. (XLSX 444 kb)

Additional file 7: Flux distributions from the MitoCore model when simulating maximum ATP production with varying proton leak through the reaction representing UCP2. (XLSX 245 kb)
